# London as a Health Centre

**Published:** 1917-08-25

**Authors:** 


### LONDON AS A HEALTH CENTRE.

### By a Social Worker.

The report for 1916 presented by Dr. W. H. Hamer, in his dual capacity of Medical Officer of Health for the Administrative County of London and of School Medical Officer, is full of interest. Accurate statistics must be extraordinarily difficult to attain, and satisfactory comparisons with other years be made almost impossible by the abnormally shifting conditions which have upset all averages. The depletion of the medical staff, and even that of the school nursing staff, have thrown difficulties in the way not merely of developing work to meet growing needs, but of carrying out an irreducible minimum. Difficulties of food supply, the massing of men in camps, the risk to health from fatigue and exposure in the case of many people taking up unaccustomed work, with the sending home of many men from infected areas, might well evoke fears of increased mortality and illness. It is, therefore, extremely cheering to learn from Dr. Hamer that "the general condition as regards the nutrition of the mass of the people has rarely, if ever, been better," and that there has been "freedom beyond the most sanguine expectation from most of the war diseases." Typhus, for instance, "obtained no footing"; there was "no serious prevalence of smallpox," and even the anxiety caused by cases of poliomyelitis has been allayed. In these cases, in Dr. Hamer's opinion, much good has been done by the wise treatment of "positive contacts," keeping them much in the open air and under observation rather than over-restricting their liberty. Excellent results were also obtained from the notifying of smallpox contacts by military to civil authorities.

### The Flea as Scarlet-Fever Carrier.

The low prevalence of scarlet fever is still more a matter for congratulation to all persons who in different ways have been occupied in raising the whole standard of cleanly living. The flea curve and scarlet fever curve, which have now for eight years been constructed, continue to show so remarkable a correspondence that some connection between the two would seem to establish the flea as a carrier of this disease. On the other hand the low prevalence of diarrhœa, which in hot summers is fatal to so many children, and the lessened mortality from diphtheria as well as scarlet fever, must in part be attributed to the wetness of the year. Dwellers in London have reason to know how much throat trouble makes itself felt when the central summer months are persistently dry and hot, even in seasons when street watering is more thorough and cleansing perhaps more effective than in war-time. We can agree with Dr. Hamer that much water has metaphorically as well as literally flowed under London Bridge during the last few years, and that we owe much to "the gradually accumulated effect of sanitary precautions worked out during the last sixty or seventy years by the health service of the country." His conclusions, therefore, offer at once an encouragement and a warning, encouragement to persevere in every kind of effort, both professional and voluntary, which can not merely reduce mortality tables, but raise the whole average of healthy life, and a warning that we shall be taking liberties with Providence if we assume that certain specially favourable conditions will establish themselves as normal and abiding. There will be trying seasons, medical staffs are depleted to the point of danger, and, in the case of smallpox, public opinion requires educating if the child population is to be fitly protected. Moreover, as regards London, the depletion of sanitary staffs begins to cause anxiety. Representations upon the delay in removal of house refuse were actually made by the Council to the Local Government Board. There is no doubt that help to the authorities in this matter is becoming a duty of citizenship. Much could be done by the drying and burning of many things which are thrown into dustbins. There can be no doubt that the number of small pet dogs should be largely reduced, whilst the provision of earth-boxes and more use of the gardens of houses and of our squares when the animals are taken out would save the pavement from defilements which increase flies and are a positive danger to people walking after dark. The need for improved sanitation is emphasised by Dr. Hamer's statistics of population, which show that natural increase, plus an inward movement from outlying suburbs, are tending to overcrowd still further the county of London.

### The War's Effect on the Death-Rate.

A curious anomaly is found in the fact that, with actually fewer deaths, the death-rate of London stands higher. This need not cause anxiety, since the reason is stated to be the withdrawal of so many men of military age, a period of low mortality. The effects of measles and influenza are unfortunate; as to the former it may well be hoped that the circulation among poor families of literature and other efforts to secure proper nursing will show an improvement next year. Spade-work has been done, and it is very satisfactory to learn that not more than 10 per cent. of the decrease of mortality at ages 0-1 can be accounted for by decline of the birth-rate. Dr. Hamer thinks that the cool summer and restrictions on the sale of intoxicants are responsible for most of the improvement. We should like to attribute more to efforts for pre-natal care, mother-schools, the improved midwifery service, and the lessening of extreme poverty. Certainly the diminution in cases of suffocation in bed from 106 in the first quarter of 1914 to 41 in the fourth quarter of 1916, or from a total of 299 to one of 147, says much for the improvement in temperance. We are glad, however, that Dr. Hamer goes on to say a good word for our much-abused system of elementary education, which has had a longer time, since the Act of 1870, to influence the women of the nation than direct efforts for mothers. A military authority has lately said that our men make good soldiers "in spite of the Board schools," and some people might incline to the view that women make better mothers "in spite of" elementary education. In view of coming developments the question is worth considering. In any case, a drop from even the record lowest rate of 91 per thousand in 1912 should urge all workers to further effort in all directions, not least in securing good hygiene teaching in the continuation classes.

The drop in civilian deaths from measles, 822, as compared with the alarming total of 2,286 in 1915 and 1,376 in 1914, is also encouraging, and is no doubt partly due to the compulsory notification both of measles and German measles, which came into force on January 1, 1916. Since we have learnt that with these diseases especially a high death-rate means a high damage-rate, we must welcome the influence of a sense of responsibility which notification urges on the uneducated. That the number of notified London cases totalled 47,470 is significant enough. Whooping-cough claimed 802 victims, though this also was a drop, and three boroughs, Holborn, Lambeth, and Greenwich, have made this complaint notifiable.

### An Opportunity for the Leisured.

All these matters affect the children. As regards the actual school work the difficulty of securing the services of enough doctors and dentists has been acutely felt. The last-named certainly points to the need for more women to qualify for the eminently suitable work of saving the children's teeth. The want of voluntary Care Committee workers was also serious, and there is much need that heads of schools and the clergy should point out to young people the valuable training for social service of many kinds given by this work. It has, moreover, the great advantage of not having to be done at fixed hours, so that many more people of limited leisure could well take it up. One result of better visiting would certainly be the larger attendance of parents at a time when the presence of a mother especially is highly desirable, the child's examination by the school doctor. Only in slightly over half the cases examined was a parent present. The need of such examinations is emphasised by the published figures. Of 256,847 children examined no fewer than 88,415 needed treatment for one or more defects. The nutrition of children generally has been better during the war than in the period immediately preceding. One interesting fact noted by the chief medical officer is that boys are more subject than girls to defective speech. As for the results of the work as a whole, Dr. Hamer is justified in calling them splendid. The upward curve in cleanliness, and in clothing and footgear and nourished condition has continued. In the matter of cleanliness both of body and head there is no doubt that unexpected visits by the school nurse are much more effective than any prepared inspection for which children can be remorselessly scrubbed and clothed with unaccustomed neatness and freshness. The superior carefulness of Jewish parents of all grades is still a reflection upon some classes of our own population. Among the poorest, imperfect efforts at cleansing seem to show that grubbiness is better than half measures. A small quantity of water and a good deal of soap used successively by different persons or children allows a terrible mixture to dry on the skin, and irritation, scratching, and skin trouble follow in natural succession. We may add that much propaganda work is wanted to persuade poor mothers to dress their children in fewer garments, especially in summer. Successive layers of torn and soiled clothing impede many small bodies like the coats of an onion. We notice that Dr. Hamer speaks of prejudice against an institution known by the candid name of "Cleansing Station," which, however, has proved its usefulness.

### Toothbrush Propaganda Needed.

People who talk with working-class folk must often notice the large amount of deafness among them, and the efforts made in London to cope with cases of dental caries with oral sepsis should presently show results. There is plenty of toothbrush propaganda still to be done. But whitening and a clean rag have advantages over the cheap toothbrush with ill-fixed and often-swallowed bristles. The condition of the average working woman's teeth points to the courageous endurance of very much preventible suffering, but also to digestive trouble and poisoning from tooth decay, which must needs affect their babies to some extent. Fulham dental centre has taken an admirable step in treating on two evenings a week the teeth of expectant and nursing mothers. Educated people may learn with surprise that of children examined by the Council's dentist, over 2,000 in number, objections to examination or treatment were raised in 214 cases. The question of vermin remains difficult. It is not satisfactory to learn that 25 per cent. of London children showed some trouble on examination, and the results of the inquiry by Mrs. Pember Reeves's Committee, published in 1913, showed that nothing less than the destruction of some dwellings could really be a cure; the tenants could not accomplish it.

While 43,450 births were notified by certificated midwives, there were 278 cases of notified puerperal fever, of which 104 proved fatal, besides 49 deaths of unnotified cases and 153 deaths from puerperal septic disease. The securing of better conditions for poor women in their confinements is for many reasons an urgent matter. It is not satisfactory to learn in this connection that the percentage of adulterated milk was higher in samples examined. The Notification of Ophthalmia Neonatorum Order resulted in 773 notifications. As only 277 of these came from practising midwives, it would seem that a wholesome sense of urgency has been established which should lessen the percentage of blindness in coming years.

